# The Novel Curcumin Derivative 1g Induces Mitochondrial and ER-Stress-Dependent Apoptosis in Colon Cancer Cells by Induction of ROS Production

**DOI:** 10.3389/fonc.2021.644197

**Published:** 2021-06-14

**Authors:** Hao Wang, Yingxing Xu, Jialin Sun, Zhongguo Sui

**Affiliations:** ^1^ Department of Medicine, Affiliated Hospital of Qingdao University, Qingdao, China; ^2^ Department of Medicine, Qingdao University, Qingdao, China

**Keywords:** 1g, colon cancer, ROS—reactive oxygen species, apoptosis, ER-stress

## Abstract

Reactive oxygen species (ROS) play an important role in cellular metabolism. Many chemotherapeutic drugs are known to promote apoptosis through the production of ROS. In the present study, the novel curcumin derivative, 1g, was found to inhibit tumor growth in colon cancer cells both *in vitro* and *in vivo*. Bioinformatics was used to analyze the differentially expressed mRNAs. The mechanism of this effect was a change in mitochondrial membrane potential caused by 1g that increased its pro-apoptotic activity. In addition, 1g produced ROS, induced G1 checkpoint blockade, and enhanced endoplasmic reticulum (ER)-stress in colon cancer cells. Conversely, pretreatment with the ROS scavenging agent N-acetyl-l-cysteine (NAC) inhibited the mitochondrial dysfunction caused by 1g and reversed ER-stress, cell cycle stagnation, and apoptosis. Additionally, pretreatment with the p-PERK inhibitor GSK2606414 significantly reduced ER-stress and reversed the apoptosis induced by colon cancer cells. In summary, the production of ROS plays an important role in the destruction of colon cancer cells by 1g and demonstrates that targeted strategies based on ROS represent a promising approach to inhibit colon cancer proliferation. These findings reveal that the novel curcumin derivative 1g represents a potential candidate therapeutics for the treatment of colon cancer cells, *via* apoptosis caused by mitochondrial dysfunction and endoplasmic reticulum stress.

## Introduction

According to cancer statistics recently published by the American Cancer Society, the incidence of human colorectal cancer (CRC) is 10.2%, with a mortality rate of 9.2%, a rise from 4th to 2nd place in the ranking of cancer (1, 2). Currently available treatments for CRC include surgical intervention, radiotherapy, and chemotherapeutic drugs. However, the mechanisms by which the treatments operate remain largely unknown (3). Therefore, new treatment strategies are required to improve survival in CRC patients.

**Figure 1:**
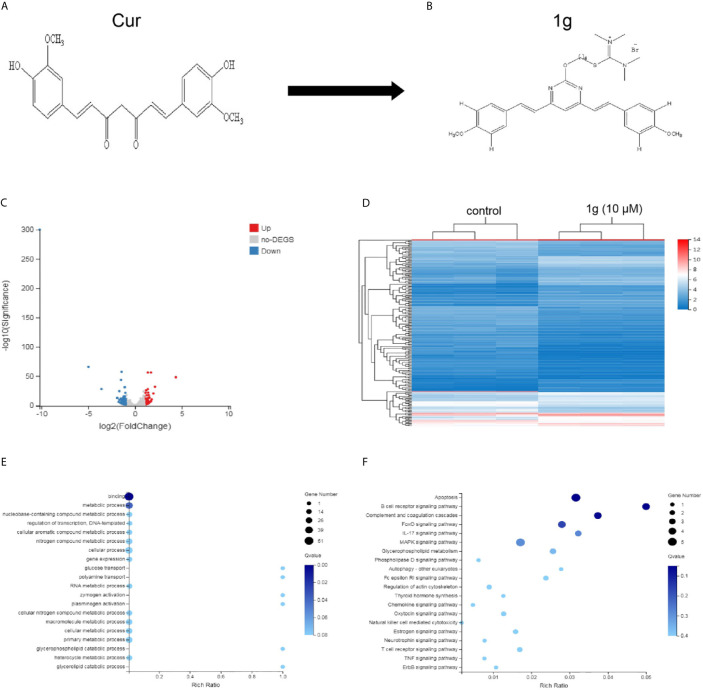
Differentially expressed gene profiles and bioinformatics analysis. HCT116 cells were exposed to 1g (10 μM) for 24 h, after which the transcriptome was sequenced. **(A, B)** The structures of Cur and 1g. **(C)** Volcano plot of differentially expressed mRNAs. **(D)** Heat map of differentially expressed mRNAs. **(E)** GO enrichment bubble map for differentially expressed mRNAs. **(F)** KEGG Pathway enrichment bubble map for differentially expressed mRNAs.

Cur (**Figure 1A**), a bioactive ingredient of *Curcuma longa* (turmeric), is a well-known cancer-preventative agent with no significant side effects at a therapeutic dose. Over the past decade, curcuma’s anti-cancer properties have been well documented in a variety of cancer types, including lung, breast, pancreatic, and prostate cancer (4–6). In addition, multiple studies have demonstrated that Cur has anti-proliferative and anti-carcinogenic properties in a variety of cell lines and animal models, partly because it blocks the cell cycle, induces apoptosis, and inhibits the proliferation and metastasis of tumor cells (7–9). Various studies have demonstrated the chemoprophylactic and antitumor effects of Cur in CRC *in vivo* (10–14). Despite its safety and extensively documented pharmacological effects, the utilization of Cur is challenging due to its extremely low water solubility, instability, and poor oral bioavailability (15, 16). A variety of strategies have been studied to overcome the limitations of Cur, including chemical modification.

ROS are highly active oxygen radicals that are mainly derived from NADPH oxidase in the mitochondria (17). It has been reported that ROS and tumor biology are inherently intertwined (18). Over recent years, this laboratory has investigated the chemical modification of Cur to create novel molecules that are more chemically stable and more pharmacologically powerful than Cur. Previously, a range of pyrimidine-substituted curcumins was synthesized and evaluated as a treatment against a variety of cancer cells (19). One compound: (E,E)-2-(4-(4,6-bis(4-methoxystyryl)pyrimidin-2-yloxy)butyl)-1,1,3,3-tetramethylisothiouronium hydrobromide (**Figure 1B**) exhibited excellent chemical stability combined with anti-cancer properties. Here, RNA-sequencing was conducted to identify differentially expressed mRNAs in a colon cancer cell line (HCT116) treated with the Cur analog 1g and found that those mRNAs identified were closely related to cell apoptosis. Subsequently, the anticancer mechanism and the effectiveness of 1g against colon cancer cells were explored. The results indicate that the anticancer mechanism of action of 1g was possibly *via* the induction of intracellular ROS generation, leading to a change in mitochondrial membrane potential, cell cycle arrest, activation of ER-stress, ultimately leading to cell apoptosis. The present study not only found that 1g inhibited the activity of colon cancer cells to a greater extent than Cur, but also revealed that overproduction of ROS is an important factor in the treatment of CRC.

## Methods

### Antibodies and Reagents

The following antibodies were used: GADPH (#2118), anti-CHOP (#2895), anti-GRP78 (#3177), anti-cleaved caspase 9 (#7237), anti-cleaved caspase 3 (#9661), anti-cleaved PARP (#5625), anti-Bax (#5023), anti-Bcl-2 (#3498), anti-Cytochrome c (#4272), anti-PERK (#5683), anti-p44/42 MAPK (Erk1/2) (#9102), anti-phospho-p44/42 MAPK (Erk1/2) (#9101), anti-Cyclin D1 (#2978), anti-phospho-Rb (#8516), anti-IRE1α (#3294), and anti-p21 (#2947), anti-mouse IgG-HRP (#7076), and anti-rabbit IgG-HRP (#7074) were purchased from Cell Signaling (Beverly, MA, USA); anti-Phospho-EIF2 alpha (AF3087) and anti-Phospho-PERK (Thr982) (DF7576) were sourced from Affinity Biosciences Ltd.; anti-ATF4 (0835-1-AP) was obtained from Proteintech (Wuhan Sanying) and anti-p53 (ab26) was acquired from Abcam (Cambridge, UK).

Other reagents used in the present study were: 1g (>98% purity, HPLC) prepared in our laboratory. Cur and DMSO were obtained from Sigma-Aldrich (St. Louis, MO, USA). Cell Counting Kit-8 (CCK-8), N-acetylcysteine (NAC; ROS scavenger), and GSK2606414 were purchased from MedChemExpress (Monmouth Junction, NJ, USA). Antibody diluent and Restore™ PLUS Western Blot Stripping Buffer were purchased from ThermoFisher Scientific (Shanghai, China).

### Cell Culture

The human colon cancer cell lines HCT116 and HT29 were obtained from the American Type Culture Collection (ATCC). SW480 and LOVO were obtained from the Cell Resource Center, Shanghai Institute of Life Sciences, Chinese Academy of Sciences. HCT116 and HT29 cells were maintained in Dulbecco’s modified Eagle’s medium (DMEM; BI, New York, NY, USA), and SW480 in Roswell Park Memorial Institute (RPMI)-1640 medium (BI). LOVO cells were maintained in DMEM/F-12 (Dulbecco’s Modified Eagle Medium/Nutrient Mixture F-12). The passage number of all the above cell lines was between 20 and 30. The medium was supplemented with 10% fetal bovine serum (FBS; Biological Industries, Israel) and 1% penicillin G/streptomycin sulfate. Cells were maintained at 37°C in a humidified incubator within 5% CO_2_.

### Transcriptome Gene Sequencing

Untreated HCT116 cells and those treated with 1g for 24 h (n = 3) underwent transcriptomic analysis by Gooal Gene Technologies Ltd. (Wuhan, China). Briefly, total RNA was processed by mRNA enrichment and rRNA removal. The RNA was fragmented with interrupt buffer, and random N6 primers were reverse transcribed, from which double-stranded cDNA was synthesized. The resultant double-stranded DNA ends were flattened and phosphorylated at the 5’-ends. A “sticky” end was formed at the 3’ end from which an ‘A’ protruded to which a bubblelike junction with a ‘T’ was connected. The ligands were amplified by PCR using specific primers. The PCR product was thermally denatured into a single strand, which was cycled with a bridge primer to obtain a single-stranded circular DNA library which was finally machine sequenced. The process is detailed in a flowchart in **Additional File 1**.

### Cell Proliferation Assay

Cell proliferation was measured using a CCK-8 assay. Briefly, 1g-treated cells were plated at 5 × 10^3^ cells per well in 96-well culture plates and cultured for 12, 24, and 48 h. A 10 µl aliquot of CCK-8 reagent was added to each well then incubated at 37°C for 3 h. The absorbance at 450 nm of each well was recorded using a microplate reader (Tecan Austria GmbH 5082, Austria) (n = 5).

### Flow Cytometry Analysis

**Cell apoptosis analysis.** Cells were seeded in six-well plates (5 × 10^5^ cells/well). After incubation with specified drug treatments, the cells were suspended in Annexin V binding buffer then incubated with Annexin V–FITC and PI in accordance with the manufacturer’s instructions (BD Biosciences, Bedford, MA, USA), prior to flow cytometry analysis (n = 3).

**Cell cycle analysis.** Cells were treated with 1g or Cur for 18 h, collected, fixed, and permeabilized overnight in 75% ethanol overnight at −20°C. All samples were washed twice with PBS and then incubated with PI and RNase at room temperature for 30 min. The results were analyzed using Modifit software (n = 3).

**ROS measurement.** ROS levels were determined using a dichloro-dihydrofluorescein diacetate (DCF-DA) probe. After treatment, the cells were collected and incubated with 20 μM DCF-DA for 30 min. After washing twice in PBS, the fluorescence intensity of DCF was measured by flow cytometry. The results were analyzed using Flowjo v10 software (n = 3).

**Mitochondrial membrane potential (ΔΨm) evaluation.** Following treatment with 1g or Cur for 24 h, the cells were incubated with a JC-1 fluorescent probe (Elabscience, Wuhan, China) in a 5% CO_2_ humidified incubator at 37°C for 20 min. The cells were then washed twice with a staining buffer and analyzed using a TMFC500 flow cytometer (Beckman Coulter, CA, USA) (n = 3).

### Western Blotting

Cells and tissues were collected in RIPA buffer (50 mM Tris, 10 mM EDTA, 1% v/v Triton-X100), and supplemented with PMSF protease inhibitor and phosphatase inhibitor. The cells and tissues were then sonicated (8 bursts of 12 s, 4°C, 100 W, using a Labsonic sonicator, Hielscher, Teltow, Germany), and the protein concentration of the samples determined using a BCA protein assay. For each sample, the same quantity of protein (35 μg) was added to an appropriate volume of 5× sample buffer (Beyotime Biotechnology, Shanghai, China), and then heated until boiling. Each sample was then separated on a 10–12.5% SDS-polyacrylamide gel (PAGE) after which they were transferred to PVDF membranes using a Bio-Rad electro-transfer system (Bio-Rad Laboratories, Munich, Germany). Each membrane was then hybridized with a primary antibody overnight on a shaking table at 4°C, washed three times with TBST buffer (3 × 10 min), then incubated with the corresponding secondary antibody at room temperature for 1 h. Finally, Immobilon Western chemiluminescent HRP matrix (Millipore, Burlington, MA, USA) was added to develop the membrane. The positive bands were vizualized using an Infrared Imaging System (LI-COR Biosciences, Lincoln, NE, USA), and the density of the digital images measured using ImageJ software (NIH, Bethesda, MD, USA, ver.1.52a) and expressed as a proportion of the GAPDH loading control (n = 3).

### Quantitative Real-Time PCR Analysis

qRT-PCR was used to measure the expression of GRP78 and CHOP. Total RNA was extracted using Trizol reagent (Invitrogen; Life Technologies Corporation, Grand Island, NY, USA) in accordance with the manufacturer’s instructions. A 1μg quantity of RNA was reverse transcribed using a PrimeScript RT reagent kit (Takara, Tokyo, Japan). The cDNA was diluted (2 µl) and selectively amplified using a real-time PCR with SYBR Green I (Takara) and specific primers. The sequences of human CHOP, GRP78, and GAPDH were obtained from previously published articles (20). The samples were amplified using a Roche LightCycler 480II (Switzerland). The relative quantity of each mRNA was calculated using a comparative method (2^−△△Ct^) against a GAPDH endogenous control (n = 3).

### Colon Cancer Xenografts in Nude Mice

All animal husbandry and experimental procedures were approved by the Animal Research Ethics Committee of the Affiliated Hospital of Qingdao University in Shandong Province. Four 6-week-old female Balb/c-nu/nu mice were obtained from the Beijing Viton Lihua Experimental Animal Technology Co. Ltd. The mice were maintained at an ambient temperature of 20–25°C, in specific pathogen-free ventilation chambers at 45–50% relative humidity, in a 12 h light-dark cycle. The mice were adapted to the environment for 7 days prior to experimentation and received sterilized food and water *ad libitum*. Suspensions of HCT116 cells were injected subcutaneously into each mouse (at a cell density of 5 × 10^6^ in 150 μl PBS). When the tumor volume had reached approximately 150 mm^3^, the mice were randomly divided into control and treatment groups (n = 6). The groups were: control group with vehicle; 1g group (40 mg/kg); 1g group (20 mg/kg), and Cur group (40 mg/kg). Each treatment group received an intraperitoneal injection once per day. The mice were monitored for 14 days during treatment. Bodyweight and tumor volume were measured every 2 days. The tumor volume was calculated using the formula: ([width]^2^×[length]/2). Moreover, weight was recorded throughout the experiment.

### HE Staining

Transplanted tumor tissue biopsies were embedded in paraffin, sectioned, then processed in xylene I for 20 min, xylene II for 20 min, ethanol I for 5 min, anhydrous ethanol II for 5 min, and 75% alcohol for 5 min prior to washing in water. Sections were stained with hematoxylin for 3–5 min, rinsed with tap water, differentiated, again rinsed with tap water, immersed in basic bluing reagent prior to rinsing in running water. Sections were then dehydrated through an 85–95% gradient of alcohol for 5 min respectively, then stained in eosin solution for 5 min. The sections were placed in anhydrous ethanol I for 5 min, anhydrous ethanol II for 5 min, anhydrous ethanol III for 5 min, dimethyl I for 5 min, xylene II for 5 min for clearing, then sealed in neutral rubber. Finally, the sections were examined by light microscopy and images were acquired then analyzed (n = 3).

### Statistical Analysis

GraphPad InStat 8.0 software (GraphPad Software, Inc., La Jolla, CA, USA) was used for statistical analysis. The results were expressed as the means of arbitrary units ± SD. Differences were analyzed using a one-way analysis of variance followed by a Bonferroni’s post-hoc test. *P*-values <0.05 were considered significant.

## Results

### Bioinformatics Analysis of Differentially Expressed mRNAs and Microarray Data of 1g-Treated Colon Cancer Cells

A total of 181 differentially expressed mRNAs were identified in the HCT116 cells after exposure to 1g (10 μM) for 24 h. Of these, 80 were up-regulated and 101 were down-regulated compared with the control group. From the results, a volcano plot was created (**Figure 1C**) and a heat map of the mRNA expression levels (**Figure 1D**). To determine the key factors that inhibit colon cancer cells by 1g, gene ontology (GO) and pathway analyses were performed. From the GO enrichment bubble map (**Figure 1E**), it is apparent that 1g inhibits HCT116 cells principally *via* regulation of the metabolic processes related to adhesion, biological regulation, cellular processes, and catalytic activity. Enrichment was conducted from information in the KEGG database to identify pathways that mediate significant change to the function of the differentially expressed genes, identifying 20 significant pathways associated with those genes that may play a key role in the processing of HCT116 cells by 1g (**Figure 1F**). Interestingly, of these, the apoptotic pathway was the most significant. Therefore, we further explored the effects and mechanisms of 1g on apoptosis in HCT116 cells.

### The Cur Derivative, 1g, Inhibits Colon Cancer Cell Proliferation

**Figure 2:**
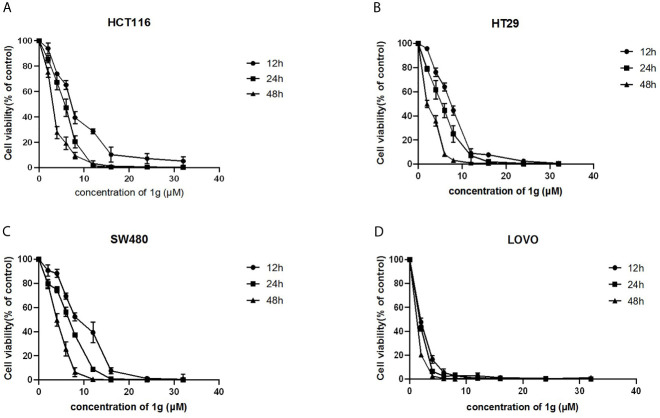
1g inhibited the proliferation of colon cancer cells. **(A)** HCT116, **(B)** HT29, **(C)** SW480, and **(D)** LOVO cells were treated with 1g (2–32 μM) for 12, 24, and 48 h. The inhibition effect of 1g on cell proliferation was dose-dependent and time-dependent (n = 5).

Using a CCK-8 assay, the effect of 1g on the proliferation of HCT116, HT29, SW480, and LOVO colon cancer cell lines at increasing concentrations for 12, 24, or 48 h, was investigated. The results indicated that 1g significantly inhibited the proliferation of the four different colon cancer cell lines in a time and dose-dependent manner (**Figures 2A–D**). However, the activity of normal CHO fibroblasts was not affected by 1g (10 μM), as reported previously (19). This suggests that 1g displays the similar safety and stability as Cur but is more selective against colon cancer cells.

### 1g Induces Apoptosis and Cell Cycle Arrest in Colon Cancer Cells

**Figure 3:**
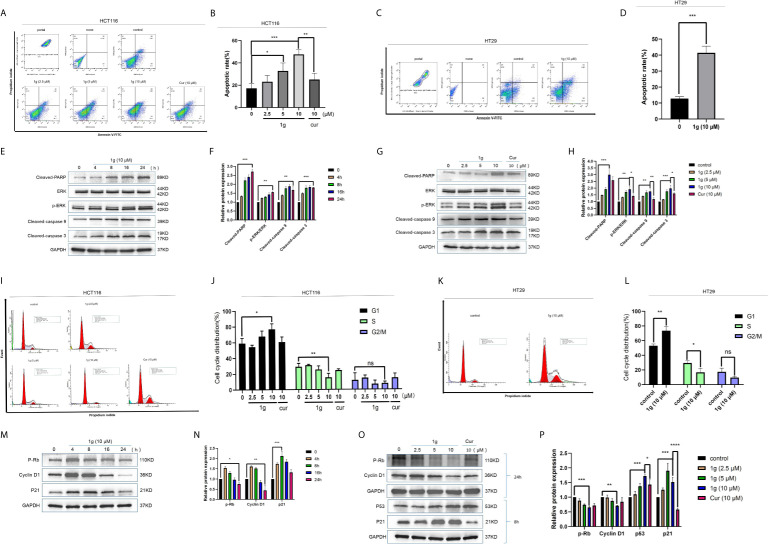
1g induced colon cancer apoptosis and inhibited cell cycle arrest in the G1 phase. **(A, B)** Flow cytometry was used to detect the induction of apoptosis of HCT116 cells after treatment with 1g or Cur for 24 h. **(C, D)** Flow cytometry was used to detect the induction of apoptosis of HT29 cells after treatment with 1g for 24 h. **(E, F)** The number of apoptotic cells was determined using GraphPad Prism software. **(C)** Levels of apoptotic proteins were evaluated in HCT116 cells treated with 1g (10 μM) over varying durations. **(G, H)** Protein levels evaluated by Western blot analysis of 1g or Cur-treated HCT116 cells for 24 h. **(I, J)** Flow cytometry was used to determine the induction of cell cycle arrest in HCT116 cells after treatment with 1g or Cur for 24 h. **(K, L)** Flow cytometry was used to determine the induction of cell cycle arrest in colon HT29 cells after treatment with 1g for 24 h. **(M, N)** Expression of relative protein levels of the cell cycle determined by Western blot analysis after treatment with 1g (10 μM) over varying durations. **(O, P)** Protein levels evaluated by Western blot analysis of 1g or Cur-treated HCT116 cells for the time indicated. Data represent means ± SD. Statistical significance was determined by one-way analysis of variance (ANOVA) followed by a Bonferroni’s *post-hoc* test. Statistical significance indicated as *P < 0.05, **P < 0.01, ***P < 0.001, ****P < 0.0001. ns indicates that the comparison was not statistically significant.

To confirm that the apoptosis caused by 1g was consistent with transcriptomic sequencing, HCT116 cells were further treated with 1g and Cur for 24 h then analyzed after Annexin V-FITC/PI double staining by flow cytometry. The number of apoptotic colon cancer cells was positively correlated with the concentration of 1g, and the number of apoptotic HCT116 cells was significantly greater when treated with 10 μM 1g than with 10 μM Cur (**Figures 3A, B**). 1g was also able to induce apoptosis in HT29 cells (**Figures 3C, D**). In addition, the apoptosis-related proteins cleaved-PARP, p-ERK, cleaved-caspase 9, and cleaved-caspase 3 were measured by Western blotting. HCT116 cells were treated with 1g (10 μM) during cell culture (0–24 h), followed by DMSO, 1g or Cur for 24 h. The results demonstrate that the protein expression levels increased after treatment with 1g in a time and dose-dependent manner (**Figures 3E–H**). Cyclin is closely involved in the checkpoint control mechanism and represents the signal to undergo apoptosis (21). Flow cytometric analysis of PI stained cells was also conducted to ascertain whether 1g induced HCT116 cell cycle arrest. HCT116 cells undergoing exponential growth were treated with 1g or Cur for 24 h then analyzed by flow cytometry. Interestingly, after treatment with 1g (10 μM), the number of cells in the G1 phase increased significantly, the number in the S phase decreased significantly while no significant change in number was observed in the G2/M phase (**Figures 3I–L**). These observations are different from those previously reported for Cur treatment of colon cancer cells, which were observed to undergo cycle arrest in the G2/M phase (8). Subsequently, the colon cancer cells were treated with 1g (10 μM) for up to 24 h. The expression levels of p-Rb and Cyclin D1 were greatest at 4 h and then decreased, while the expression levels of p21 were highest at 8 h (**Figures 3M, N**). In addition, the expression levels of P-Rb and Cyclin D1 were measured in HCT116 cells treated with increasing concentrations of 1g or Cur at 24 h. The expression levels of p21 and p53 were measured at 8 h. P-Rb and Cyclin D1 expression decreased with increasing 1g concentration, while the expression levels of p21 and p53 increased. This is consistent with the regulation of the cell cycle by these proteins (**Figures 3O, P**).

### 1g Induces Changes in Mitochondrial Membrane Potential in Colon Cancer Cells

**Figure 4:**
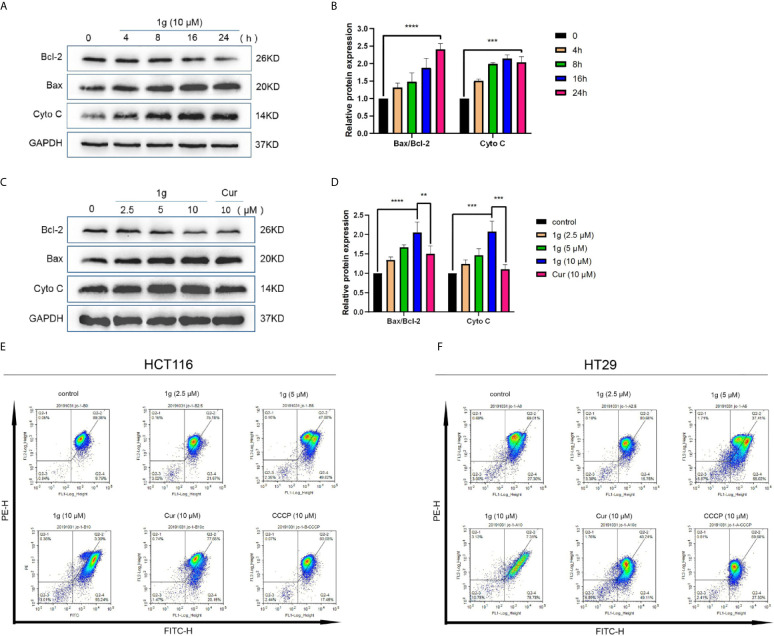
1g changes the mitochondrial membrane potential in colon cancer cells. **(A, B)** HCT116 cells were treated with 1g (10 μM) over varying durations. **(C, D)** Protein levels evaluated by Western blot analysis of 1g or Cur-treated HCT116 cells for 24 h. **(E)** Mitochondrial membrane potential was analyzed by flow cytometry in HCT116 cells treated with 1g or Cur for 24 h (n = 3). **(F)** Mitochondrial membrane potential was analyzed by flow cytometry in HT29 cells treated with 1g for 24 h (n = 3). Data represents means ± SD. Statistical significance was determined by one-way analysis of variance (ANOVA) followed by a Bonferroni’s *post-hoc* test. Statistical significance indicated as **P < 0.01, ***P < 0.001, ****P < 0.0001.

It is well-known that many cytotoxic stimuli, including chemotherapeutic drugs, induce apoptosis *via* endogenous pathways with which mitochondria are involved (22, 23). Apoptosis-related gene expression was assessed to test the hypothesis that 1g operates similarly, finding that Bax and Cyto C expression levels increased and anti-apoptotic Bcl-2 expression levels decreased after the treatment of HCT116 cells with 1g, in a time and dose-dependent manner (**Figures 4A–D**). As the ratio of Bax/Bcl-2 expression increased, mitochondrial membrane dysfunction and permeability increased (24). The mitochondrial membrane potential of HCT116 and HT29 cells treated with 1g or Cur for 24 h was found to have undergone a clear change (**Figures 4E, F**).

### 1g Induces Colon Cancer Apoptotic Death by Evoking ROS Generation and Transduction of the ER Stress-Related Cell Death Pathway

**Figure 5:**
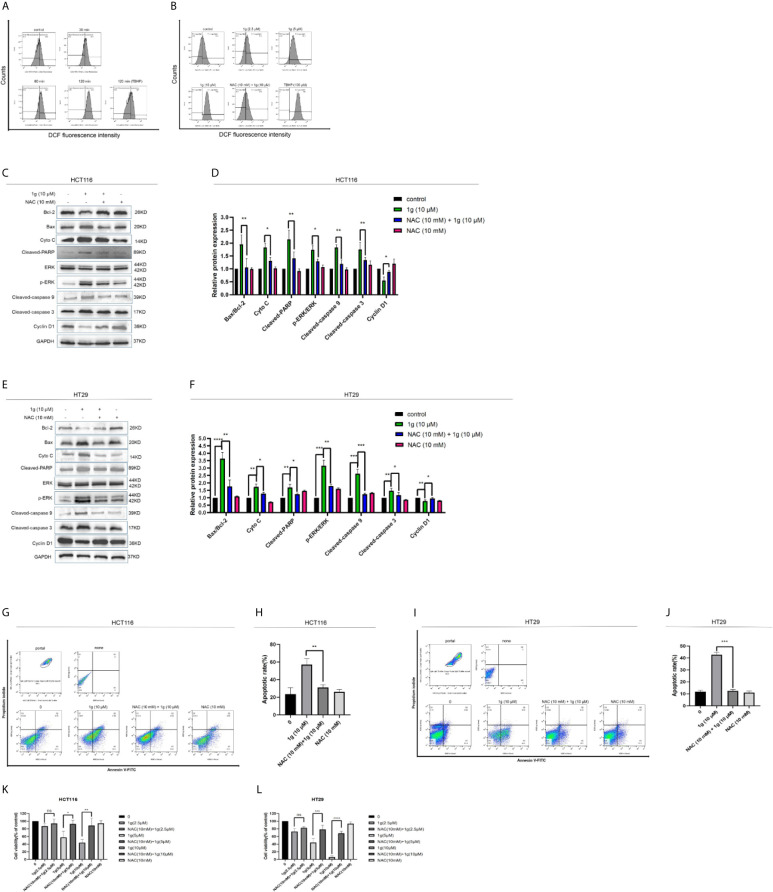
Cytotoxicity of 1g toward colon cancer cells depends on the production of intracellular ROS. **(A)** Flow cytometric analysis of intracellular ROS generation induced by 1g over increasing durations measured in HCT116 cells after staining with DCFH-DA (10 μM). **(B)** HCT116 cells pre-incubated with NAC (10 mM) for 1 h prior to exposure to 1g (10 μM) for 8 h. **(C, D)** HCT116 cells pretreated with NAC (10 mM) for 1 h, then treated with 1g (10 μM) for 24 h. Apoptotic proteins and cyclins measured by Western blotting. **(E, F)** HCT116 cells pretreated with NAC (10 mM) for 1 h, then treated with 1g (10 μM) for 24 h. **(G, H)** Flow cytometry was used to detect the induction of apoptosis of HCT116 cells. **(I, J)** Flow cytometry was used to detect the induction of apoptosis of HCT116 cells. **(K, L)** HCT116 and HT29 cell activity measured using a CCK-8 assay. Data represent means ± SD. Statistical significance was determined by one-way analysis of variance (ANOVA) followed by a Bonferroni’s *post-hoc* test. Statistical significance is indicated as *P < 0.05, **P < 0.01, ***P < 0.001, ****P < 0.0001. ns indicates that the comparison was not statistically significant.

**Figure 6:**
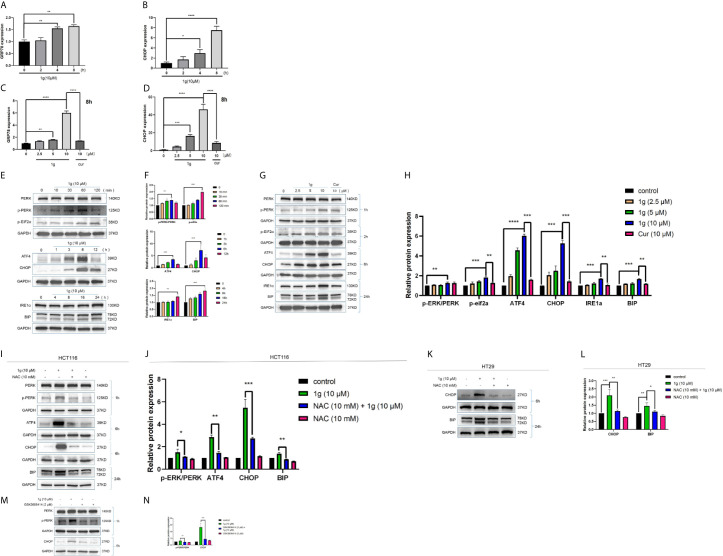
ER-stress is involved in 1g-induced apoptosis of colon cancer cells. **(A, B)** GRP78 and CHOP mRNA levels of HCT116 cells were measured after treatment 1g (10 μM) from 0 to 8 h. **(C, D)** GRP78 and CHOP mRNA levels of HCT116 cells were measured after treatment with various concentrations of 1g ranged from 0 to 10 μM for 8 h. **(E, F)** ER-stress protein levels were measured by Western blot analysis of HCT116 cells treated with 1g (10 μM) for various durations. **(G, H)** Protein levels were measured by Western blot analysis of HCT116 cells treated with 1g or Cur for the duration indicated. **(I, J)** Expression of proteins measured by Western blot analysis of HCT116 cells pre-incubated with 10 mM NAC for 1 h prior to treatment with 1g (10 μM) for the duration indicated. **(K, L)** Expression of proteins measured by Western blot analysis of HT29 cells pre-incubated with 10mM NAC for 1 h prior to treatment with 1g (10 μM) for the duration indicated. **(M, N)** Expression of PERK, p-PERK, or CHOP determined by Western blot analysis of HCT116 cells incubated with p-PERK inhibitor (GSK2606414), after stimulation with 1g (10 μM) for 2 or 6 h. Data represent means ± SD. Statistical significance was determined by one-way analysis of variance (ANOVA) followed by a Bonferroni’s *post-hoc* test. Statistical significance is indicated as *P < 0.05, **P < 0.01, ***P < 0.001, ****P < 0.0001. ns indicates that the comparison was not statistically significant.

As is well known, mitochondria are the principal source of ROS in eukaryotic cells. The destruction of mitochondrial function is generally related to an enhancement of the production of mitochondrial ROS (25). Excessive production of ROS can lead to DNA damage (26), endoplasmic reticulum stress (27), and apoptosis (28). To test the hypothesis that 1g was able to stimulate colon cancer cells to produce ROS, production was measured in 1g-treated cells, which demonstrated increased ROS production in a time-dependent manner (0–120 min) (**Figure 5A**). N-acetyl-l-cysteine (NAC) is an antioxidant that can eliminate all ROS subclasses (29). The ROS produced by HCT116 cells was measured after treatment with different concentrations of 1g and pretreatment with 10 mM NAC for 1 h. ROS increased with increasing concentrations of 1g, while ROS production was significantly reduced in the NAC pretreatment group (**Figure 5B**). To verify that the ROS produced in colon cancer cells led to cell apoptosis and cell cycle stagnation, ultimately leading to decreased cell activity, the cells were pretreated with NAC for 1 h, followed by treatment with 1g (10 μM) for 24 h. The expression of mitochondrial proteins and cycle proteins was detected by Western blotting (**Figures 5C–F**). As demonstrated by flow cytometry, NAC significantly reversed 1g-induced apoptosis of colon cancer (**Figures 5G–J**). The activity of HCT116 and HT29 cells was measured using a CCK-8 assay (**Figures 5K, L**). To further explore whether 1g (10 μM)-stimulated ROS production of colon cancer cells can cause their endogenous apoptosis by ER-stress, the gene expression of CHOP and GRP78 was quantified by RT-PCR in HCT116 cells at various times (0 to 8 h) (**Figures 6A, B**), and CHOP and GRP78 gene expression in 1g and Cur-treated HCT116 cells for 8 h (**Figures 6C, D**). The results demonstrated that the mRNA transcription of GRP78 and CHOP was time-dependent and increased with the increasing 1g concentration. The time dependency of ER-stress related protein expression was then measured by the Western blotting of PERK, p-PERK, and p-EIF2α (0–120 min); ATF4 and CHOP (0–12 h); and IRE1α and BIP (0–24 h). Maximum protein expression of p-PERK occurred at 1 h, p-EIF2α at 2 h, ATF4 and CHOP after 6 h, and IRE1α and BIP at 24 h (**Figures 6E, F**). The effect on ER-stress related protein expression of different concentrations of 1g and Cur was investigated at the specific exposure times above. The protein expression of p-EIF2α, p-PERK, CHOP, and ATF4 were increased with increasing 1g concentration, while the expression of IRE1α and BIP was greatest at 1g (10 μM) (**Figures 6G, H**). After 1 h of pretreatment with NAC, HCT116 cells were treated with 1g (10 μM) after which they were analyzed for protein expression. It demonstrated that the expression of CHOP, ATF4, and p-PERK was almost entirely blocked by pretreatment with NAC and the expression of BIP significantly reduced (**Figures 6I, J**). Similarly, in HT29 cells, NAC reversed the expression of CHOP and BIP proteins (**Figures 6K, L**). To determine whether ER-stress was the cause of 1g-induced apoptosis and death of the colon cancer cells, they were treated with the p-PERK inhibitor GSK2606414 (30). Compared with the untreated group, p-PERK was significantly reduced after 1 h pretreatment with GSK2606414 (2 μM). Similarly, the expression of proteins downstream of CHOP decreased significantly (**Figures 6M, N**). These results indicate that ER stress signal activation at least partially mediated 1g-induced apoptosis of HCT116 cells.

### 1g Significantly Inhibits HCT116 Cell Growth in a Mouse Model

**Figure 7:**
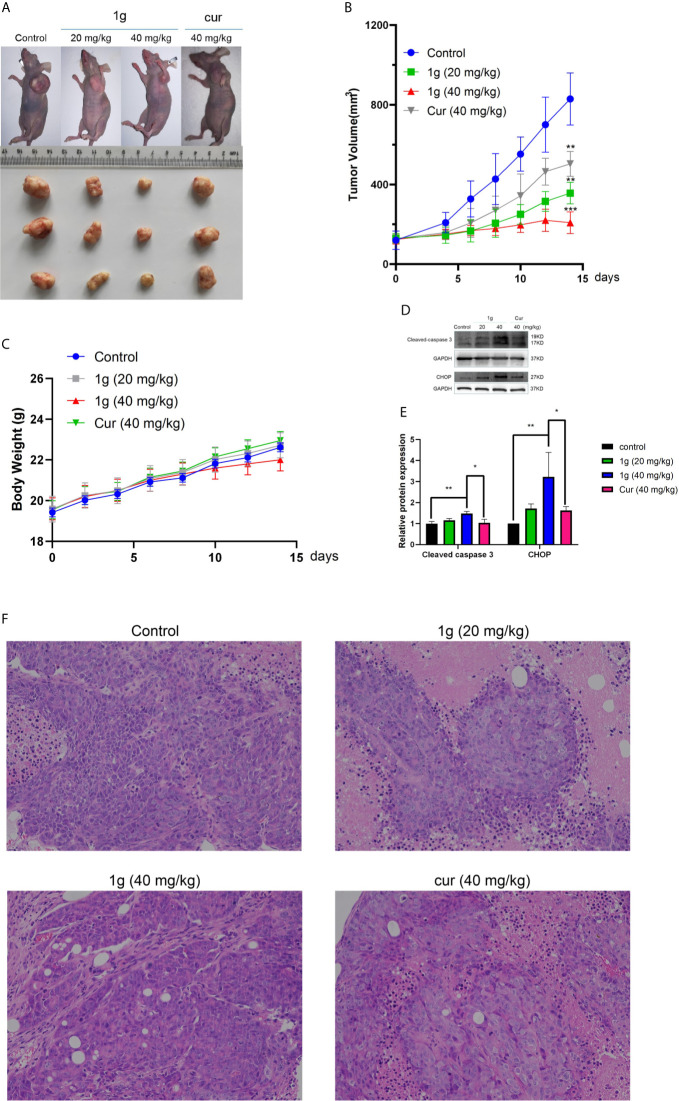
1g inhibits tumor growth in a xenograft mouse model of human colon cancer. **(A)** 7 days after HCT116 cell implantation, the mice were treated with either vehicle (control), 1g (20 mg/kg), 1g (40 mg/kg), or Cur (40 mg/kg) for an additional 14 days. The size of the tumor in each group is shown (n = 6 each group). **(B)** Representative images of tumors in each treatment group. **(C)** Weight of Balb/c mice (n = 6). **(D, E)** Cleaved caspase 3 and CHOP levels evaluated in 1g-treated and Cur-treated xenografts. Relative density measurements correspond to Western blot band intensities normalized to an internal control (n = 3). **(F)** HE staining of tumor tissue treated with 1g and curcumin. Data represent means ± SD. Statistical significance was determined by one-way analysis of variance (ANOVA) followed by a Bonferroni’s *post-hoc* test. Statistical significance is indicated as *P < 0.05, **P < 0.01, ***P < 0.001.

To verify the anti-tumor effect of 1g *in vivo*, HCT116 cells were injected into mice to establish an *in vivo* tumor xenograft model. The results demonstrated that the volume of the resultant tumors in mice treated with saline increased in a time-dependent manner, while both 1g and Cur displayed antitumor effects (**Figure 7A**). The size of the tumors in mice of the 1g group was distinctly smaller than those in the mice of the Cur and control groups (**Figure 7B**). Furthermore, all mice survived 14 days of treatment and were approximately the same weight. The results indicate that 1g was shown to be safe at the specified dose (**Figure 7C**). Western blotting and immunohistochemical analysis indicated that 1g significantly enhanced the expression of cleaved-caspase 3 and CHOP (**Figures 7D, E**). From the results of hematoxylin-eosin (HE) staining, a large number of pathological mitotic figures were observed in the tumor tissue treated with 1g (**Figure 7F**). These data are consistent with the results of 1g in anti-colon cancer cell proliferation experiments *in vitro*. In addition, 1g exhibited stronger anti-tumor effects *in vivo* than Cur at the same dose.

## Discussion

Cur is the biologically active ingredient in turmeric, known since ancient times for its medicinal properties (31). Cur is thought to induce apoptosis in all types of cancer cell (32–34). Over the past decade, cur has demonstrated remarkable biological properties, but its clinical use has been limited by its instability at physiological pH and low bioavailability (35, 36). Multiple studies have shown that the reason for Cur’s poor bioavailability is its early biological transformation and metabolism (37, 38). In order to overcome this limitation, Cur has been chemically modified to improve its stability and bioavailability. Interestingly, a series of isothiourea-modified compounds with important pharmacological activity, including anti-tumor properties, were produced (39–41). Based on these compounds, we introduced the positively charged isothiourea group into the pyrimidine-substituted curcumin analog. Our data indicated that 1g significantly enhanced anticancer activity compared to the unmodified compounds, in addition to its unique Golgi body localization. However, the mechanism by which 1g inhibits colon cancer growth remains unclear (19).

Here, we identified 181 differentially expressed mRNAs in HCT116 cells following exposure to 1g (10 μM) for 24 h, and additional bioinformatics analysis indicated that these differentially expressed mRNAs were closely related to cell apoptosis. As a result, we explored the effects and mechanisms of 1g on apoptosis in HCT116 cells. The data indicate that 1g reduced the activity of colon cancer cells in a time and dose-dependent manner. Furthermore, transcriptional genome sequencing revealed that it mainly induced apoptosis and death of colon cancer cells. As described above, it has been confirmed the molecular mechanism by which Cur induces apoptosis is principally through the mitochondria-dependent pathway (32, 38). Flow cytometry analysis and Western blotting revealed that 1g modified the mitochondrial membrane potential of HCT116 cells and induced apoptosis. Interestingly, the change in mitochondrial membrane potential induced by 1g activated apoptotic signals, unlike the mechanism of Cur. In addition, Western blot analysis of apoptotic proteins demonstrated that 1g effectively induced apoptosis by releasing cytochrome c and activating downstream caspase-9 and caspase-3 cascade reactions.

In recent years, increasing attention has focused on therapeutic drugs that can regulate different stages of the cell cycle in cancer cells, including G0/G1, S, and G2/M (42). Regulation by inhibition of the cell cycle may be considered a beneficial event that leads to the induction of apoptotic cell death when treating colon cancer (43). In the present study, in comparison with Cur (8), 1g induced cell blockade of the G1 phase in HCT116 cells. Thus, the results indicate that 1g caused apoptosis in colon cancer cells by blocking the cell cycle.

**Figure 8:**
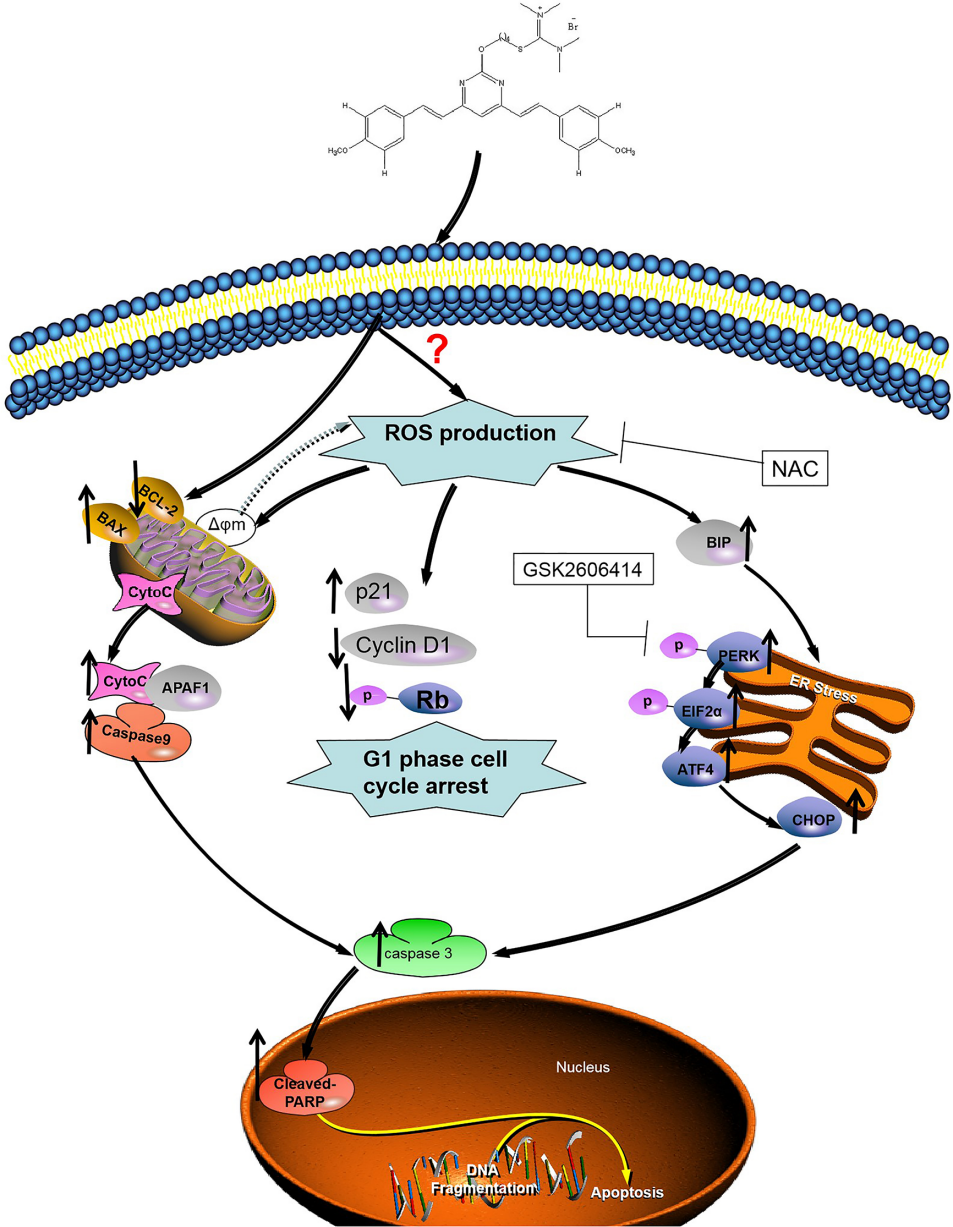
Illustration of the mechanism of inhibition of colon cancer growth by 1g. 1g causes the production of mitochondrial ROS, which then leads to cell cycle stagnation and ER-stress, resulting in apoptotic cell death.

Cur also produced ROS, leading to apoptosis in cancer cells (44, 45). As important multifaceted signaling molecules, ROS regulate multiple cellular pathways and play an important role in deciding cell fate (46). There is growing evidence that excessive oxidative stress may be an effective method of eliminating cancer cells (47). After excessive production of ROS, a number of pro-apoptotic signaling pathways, including ER-stress, are activated (48). ER-stress is a conservative cellular defense mechanism that responds to the environment within the ER (49). A number of anticancer drugs have previously been reported to induce apoptosis in cancer cells by ER-stress, such as sphenol (50), farnicol (51), or polyfiline D (52). Similarly, 1g also promoted ROS-mediated ER-stress. The unfolded protein response causes ER stress over a long period and activates the mammalian apoptosis pathway (53). During the commitment stage of ER-stress-induced apoptosis, activation of the downstream transcription factor CHOP caused by ATF-4 signaling can trigger pro-apoptotic signals, thereby triggering the specific cascade of ER-stress that results in apoptosis (54). We used Western blotting to identify the association between apoptosis and ER-stress in colon cancer cells induced by 1g. We further investigated whether apoptosis induced by 1g was related to ER stress. HCT116 cells were pretreated with PERK inhibitor. Inhibition of P-PERK by GSK2606414 significantly inhibited the expression of CHOP and p-PERK in 1g-induced HCT116 cells. Importantly, however, the inhibition of ROS production by combined NAC treatment almost completely reversed HCT116-induced apoptosis, including cell cycle blockade, mitochondrial apoptosis, and ER-stress. These results further demonstrate that ROS production plays a critical role in cell survival. The results suggest, as illustrated in **Figure 8**, that 1g can induce the production of ROS in colon cancer cells, resulting in cell cycle stagnation, a change in mitochondrial membrane potential and ER-stress, thus leading to apoptosis in colon cancer cells. Indeed, in addition to cellular effects, we demonstrated that 1g has a strong inhibitory effect on tumor growth in an HCT116 transplant tumor mouse model. Moreover, 1g displayed an excellent safety profile.

In summary, The Cur derivative 1g significantly reduced the activity of colon cancer cells and induced apoptosis of colon cancer cells *in vitro* and *in vivo* through the production of ROS. The properties of 1g should be further studied in order to develop an effective anticancer drug to treat human colon cancer. The results also suggest that activation of ROS production may be a key factor in the treatment of CRC. Nevertheless, the direct target of the 1g molecule remains unknown and the specific origin of ROS is still unclear. Current data indicate a high probability that it is generated in mitochondria, but other aspects require additional study. Therefore, we believe that the over-production of ROS following the modification of natural anticancer drugs may provide a novel strategy for anticancer treatment.

## Data Availability Statement

The datasets presented in this study can be found in online repositories. The names of the repository/repositories and accession number(s) can be found below: https://www.ncbi.nlm.nih.gov/bioproject/?term=PRJNA716135.

## Ethics Statement 

The animal study was reviewed and approved by the Ethics Committee of Experimental Animal Welfare of the Affiliated Hospital of Qingdao University (AHQU20200605).

## Author Contributions

HW and JS designed the experiments and helped write the manuscript. YX designed the experiments and checked the text of the article. ZS designed the and raised the funding for the experiments. All authors contributed to the article and approved the submitted version.

## Funding

This study was supported by grants from the National Youth Foundation of China (81903872) and the Natural Science Foundation of Shandong Province (ZR2019MH077).

## Conflict of Interest

The authors declare that the research was conducted in the absence of any commercial or financial relationships that could be construed as a potential conflict of interest.

## Abbreviations

ATCC, American Type Culture Collection; CRC, colorectal cancer; Cur, Curcumin; DMSO, dimethyl sulfoxide; ER, endoplasmic reticulum; GO, gene ontology; HE, hematoxylin-eosin; KEGG, Kyoto Encyclopedia of Genes and Genomes; NAC, N-acetyl-l-cysteine; qRT-PCR, Quantitative real-time PCR; ROS, reactive oxygen species.
