# Supplementary figures and images for: The Novel Curcumin Derivative 1g Induces Mitochondrial and ER-Stress-Dependent Apoptosis in Colon Cancer Cells by Induction of ROS Production
Source: Front Oncol. 2021 Jun 14;11:644197. doi: 10.3389/fonc.2021.644197 (PMC8236884; doi:10.3389/fonc.2021.644197)

**Supplementary Information**

**Additional file 1**


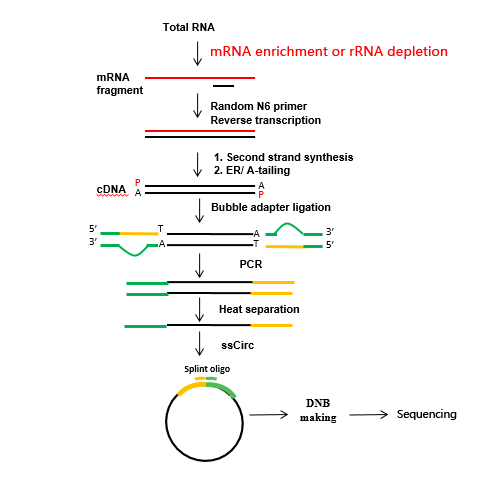

Supplement: Supplementary file 1 [file DataSheet_1.docx]
